# Inhibition of Caffeine Metabolism by *Apiaceous* and *Rutaceae* Families of Plant Products in Humans: *In Vivo* and *In Vitro* Studies

**DOI:** 10.3389/fphar.2021.641090

**Published:** 2021-04-29

**Authors:** Zeyad Alehaideb, Mohamed Sheriffdeen, Francis C. P. Law

**Affiliations:** ^1^ Department of Medical Research Core Facility and Platforms, King Abdullah International Medical Research Center, Riyadh, Saudi Arabia; ^2^ King Saud Bin Abdulaziz University for Health Sciences, Riyadh, Saudi Arabia; ^3^ Ministry of National Guard ‐ Health Affairs, Riyadh, Saudi Arabia; ^4^ Life Sciences Division, SGS Canada Inc., Mississauga, ON, Canada; ^5^ Department of Biological Sciences, Faculty of Science, Simon Fraser University, Burnaby, BC, Canada

**Keywords:** caffeine, furanocoumarin, enzyme inactivation mechanism, P450 cytochrome enzymes, chemical mixtures

## Abstract

Daily consumption of caffeinated beverages is considered safe but serious health consequences do happen in some individuals. The *Apiaceous* and *Rutaceae* families of plant (ARFP) products are popular foods and medicines in the world. We previously reported significant amounts of furanocoumarin bioactive such as 8-methoxypsoralen, 5-methoxypsoralen, and isopimpinellin in ARFP products. As both caffeine and furanocoumarin bioactive are metabolized by the same hepatic CYP1A1/2 isozyme in humans, caffeine/ARFP product interactions may occur after co-administration. The objectives of the present study were to study *in vivo* loss of caffeine metabolizing activity by comparing the pharmacokinetics of caffeine in volunteers before and after pre-treatment with an ARFP extract, study the correlation between the decrease in hepatic CYP1A2 activity and the content of furanocoumarin bioactive in ARFP extracts, characterize CYP1A2 inactivation using *in vitro* incubations containing ^14^C-caffeine, a furanocoumarin bioactive, and human liver microsomes (HLMs), and provide a mechanistic explanation for both *in vivo* and *in vitro* data using the irreversible inhibition mechanism. The study results showed pre-treatment of volunteers with four ARFP extracts increased the area-under-the-concentration-time-curve (AUC_0-inf_) ratio of caffeine in the plasma ranging from 1.3 to 4.3-fold compared to the untreated volunteers indicating significant caffeine metabolism inhibition. The increases in AUC_0-inf_ ratio also were linearly related to the effect-based doses of the furanocoumarins in the ARFP extracts, a finding which indicated caffeine metabolism inhibition was related to the content of furanocoumarin bioactive in an ARFP product. *In vitro* incubation studies also showed individual furanocoumarin bioactive were potent inhibitors of caffeine-N-demethylation; the IC_50_ for 8-methoxypsoralen 5-methoxypsoralen, and isopimpinellin were 0.09, 0.13, and 0.29 µM, respectively. In addition, CYP1A2 inactivation by individual furanocoumarin bioactive was concentration- and time-dependent involving the irreversible inhibition mechanism. The proposed irreversible inhibition mechanism was investigated further using ^14^C-labeled 8-methoxypsoralen and HLMs. The formation of ^14^C-adducts due to ^14^C-8-MOP-derived radioactivity bound to HLMs confirmed the irreversible inhibition of CYP1A2 activity. Thus, furanocoumarin bioactive metabolism in humans would result in reactive metabolite(s) formation inactivating CYP1A2 isozyme and inhibiting caffeine metabolism. Once the CYP1A2 isozyme was deactivated, the enzymic activity could only be regained by isozyme re-synthesis which took a long time. As a result, a single oral dose of ARFP extract administered to the human volunteers 3.0 h before still was able to inhibit caffeine metabolism.

## Highlights:

- A single oral dose of an ARFP extract administered to humans 3 h before is still able to inhibit caffeine metabolizing activity.
- The degree of caffeine metabolism inhibition is linearly related to an increasing effect-based dose of the furanocoumarin mixture in the ARFP extracts.
- Individual furanocoumarin chemicals inactivate CYP 1A2 enzyme in a time- and concentration-dependent manner which indicates involvement of the irreversible inhibition mechanism.
- The long inhibitory effects of ARFP on caffeine metabolism are explainable by CYP 1A2 inactivation.

## Introduction

**FIGURE 1:**
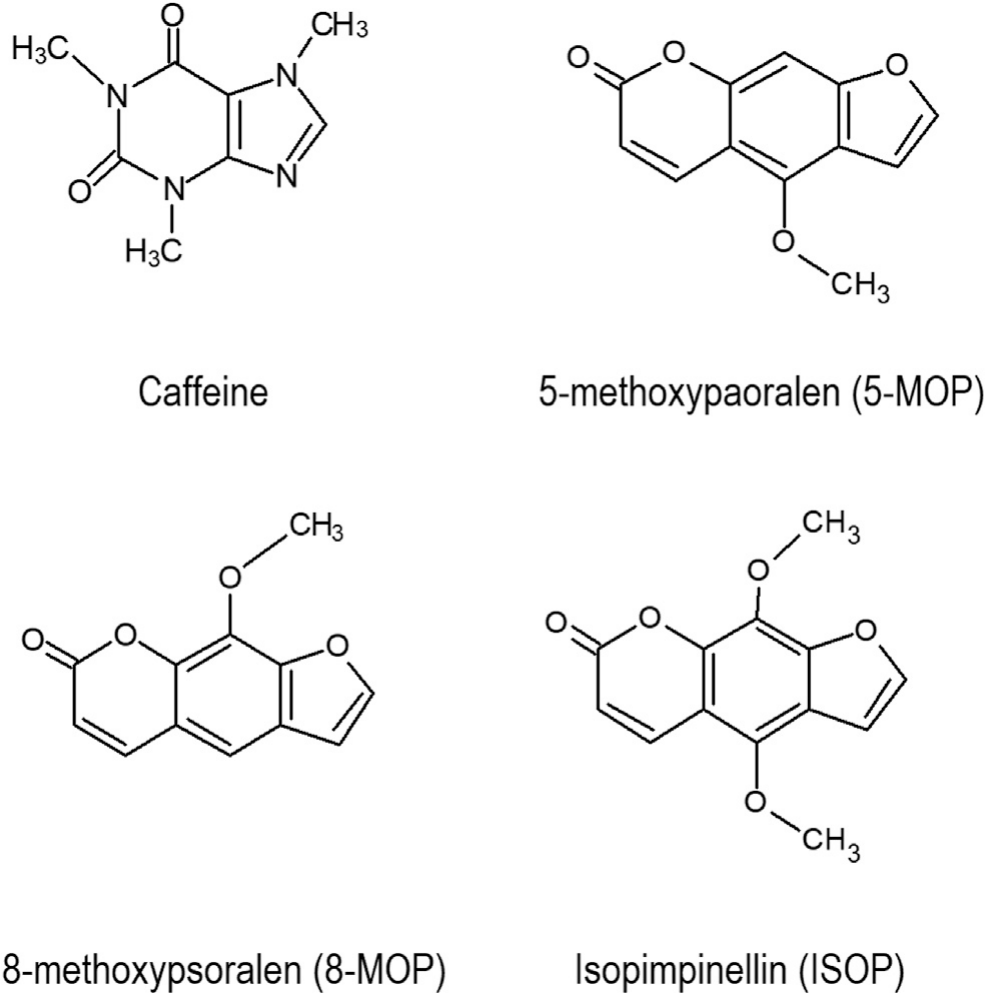
Chemical structures of caffeine and linear furanocoumarins.

Caffeine (Figure 1) is a popular human stimulant. About 100 mg of caffeine is consumed by each person daily in the form of coffee, tea and/or cocoa (FAOSTAT, 2009). The caffeine consumption level would be higher if energy drinks, fitness supplements, and caffeine-containing drugs were included in the estimation (Goldstein et al., 2010; Lipton et al., 2017). Caffeine is often used to improve physical and/or cognitive performance in humans (Glade, 2010). Using caffeine in moderation is safe, but excessive usage would lead to serious health problems such as severe cardiac arrhythmia, delirium, seizures (Nawrot et al., 2003; Akter et al., 2016; Wikoff et al., 2017), and even death (Jabbar and Hanly, 2013).

The pharmacokinetics of caffeine in humans has been studied in great details (Tang-Liu et al., 1983). Caffeine is almost completely absorbed by humans after oral administration and caffeine usually reaches the peak concentration in the blood within 1.0 h after receiving a single oral dose. Caffeine is metabolized by the hepatic cytochrome (CYP) 1A2 isozyme of humans mainly to paraxanthine (Kot and Daniel, 2008) with less than 3% of the administered dose eliminated as unchanged drug (Tang-Liu et al., 1983). The half-life of caffeine in the plasma is reported to range from 2.7 to 9.9 h (Blanchard and Sawers, 1983). Thus, the pharmacokinetics of caffeine in the plasma of humans shows significant *inter*- and *intra*-individual differences (Tian et al., 2019).

Previous studies have shown that the furanocoumarin bioactive in grapefruits (Guo and Yamazoe, 2004) and herbaceous plants (Zhuang et al., 2013) are potent CYP enzyme inhibitors. However, little is known of the inhibitory effects of the *Apiaceous* and *Rutaceae* families of plant (ARFP) products on human CYP enzymes. The ARFP products are popular foods and traditional medicines in the Middle East and Asia. In an earlier study, we screened over 29 products from the ARFP for linear furanocoumarins using high-performance liquid chromatography equipped with an ultra-violet detector (HPLC-UV), and gas chromatography with mass spectrometry (Alehaideb et al., 2017). Our results showed significant levels of 8-methoxypsoralen (8-MOP), 5-methoxypsoralen (5-MOP), and isopimpinellin (ISOP) (Figure 1) were found in 9 of the ARFP products; total linear furanocoumarins in these ARFP products ranged from 0.016 to 11.468 mg/g dry weight. It should be noted that linear furanocoumarins are chemical derivatives of psoralen whereas angular furanocoumarins are derivatives of isopsoralen which is an isomer of psoralen (Berenbaum, 1983). Also, linear furanocoumarins such as psoralen (Zhuang et al., 2013), ISOP (Kang et al., 2011), imperatorin and isoimperatorin (Cao et al., 2013), and bergamottin (Lim et al., 2005) have been identified as inhibitors of human CYP 1A2 enzyme.

The objectives of the present study were: to investigate nine selected ARFP products for the potential to inhibit caffeine metabolism by comparing the pharmacokinetics of caffeine in humans before and after pre-treatment with an ARFP extract, to determine the relationship between the area-under-the-concentration-time-curve (AUC) ratio of caffeine and the content of furanocoumarin bioactive in an ARFP extract, to study *in vitro* CYP 1A2 inactivation with incubations containing ^14^C-caffeine, a furanocoumarin inhibitor, and human liver microsomes (HLMs), and to propose an inhibition mechanism to explain the *in vivo* and *in vitro* data.

It is important to study CYP 1A2 inactivation by ARFP products in humans. Although the CYP 1A2 isozyme accounts for just 13% of the total hepatic CYP pool (Shimada et al., 1994), it plays an important role in the biotransformation of prescription drugs and pro-carcinogens. As such inactivation of CYP 1A2 by ARFP products has both beneficial and harmful health effects: the beneficial effects include the prevention of carcinogenesis by decreasing pro-carcinogen activation whereas the harmful effects include caffeine overdose as a result of caffeine/ARFP product interaction.

## Materials and Methods

### Source of Plant Products, Chemicals, and Human Liver Microsomes

#### *Apiaceae* and *Rutaceae* Families of Plant Products

We selected nine ARFP products for the present study based on the results of an earlier study (Alehaideb et al., 2017). The selected ARFP products were *Ammi majus* L. (*A. majus*) purchased from EverWilde (Fallbrook, CA), *Angelica archangelica* L. seeds (*A. archangelica*), *A. graveolens* seeds (*A. graveolens* S), *Pimpinella anisum* L. seeds (*P. anisum*), and *Ruta graveolens* L. leaves (*R. graveolens*) were purchased from Mountain Rose (Eugene, OR). *Apium graveolens* L. flakes (*A. graveolens* F) and *Petroselinum crispum* (Mill.) Fuss leaves (*P. crispum*) were purchased from A1SpiceWorld (Glen Head, NY). *Angelica pubescens* Maxim. roots (*A. pubescens*) purchased from Spring Wind (San Francisco, CA), *Cnidium monnieri* (L.) Cusson (*C. monnieri*) were purchased from Health and Wellness House (Duncan, BC).

These nine ARFP products were authenticated by the suppliers and certified to be free of pesticides and preservatives. Further authentication was performed by measurement of furanocoumarin levels chromatographically in the aforementioned ARFP as seen in Table 1. The furanocoumarins in the aqueous extracts were separated using a gradient program consisting of acetonitrile and water and detected by UV set at 310 nm for 40 min. The measured levels of linear furanocoumarins in ARFP were compared with previously published levels in same plant species (Alehaideb et al., 2017). Voucher samples were kept for future reference and certificates are available upon request.

**TABLE 1:** The amounts of linear furanocoumarins found in various ARFP products.

Botanical name	Plant part	Linear furanocoumarins content (µg/g) dry weight^a^		
		8-MOP	5-MOP	ISOP
*Ammi majus*	Seed	3213.5 ± 219.7	717.2 ± 6.5	7537.2 ± 1492.9
*Angelica archangelica*	Root	651.4 ± 51.8	392.5 ± 208.0	606.0 ± 131.9
*Angelica pubescens*	Root	25.6 ± 0.0	32.5 ± 20.0	n.d^b^
*Apium graveolens*	Seed	21.0 ± 4.4	16.9 ± 6.0	236.5 ± 22.4
*Apium graveolens*	Flakes	12.1 ± 1.6	243.2 ± 39.7	9.5 ± 0.9
*Cnidium monnieri*	Fruit	707.1 ± 78.8	1788.1 ± 152.3	466.8 ± 95.4
*Petroselinum crispum*	Leaves	n.d^b^	34.4 ± 10.6	n.d^b^
*Pimpinella aniseum*	Seed	15.8 ± 5.8	n.d^b^	n.d^b^
*Ruta graveolens*	Leaves	1342.4 ± 135.7	534.0 ± 120.6	294.9 ± 49.5

^a^ Results are expressed as mean ± SD from minimum three aqueous extractions.

^b^ n. d. = not detected.

#### Chemicals

Benzotriazole (99.0%), caffeine (≥99.0%), ethyl acetate (≥99.7%), 8-MOP (≥98.0%), 5-MOP (99.0%), and β-nicotinamide adenine dinucleotide phosphate reduced form (NADPH) (≥97.0%), were obtained from Sigma-Aldrich (St. Louis, MO). Methanol (≥99.9%) and acetonitrile (≥99.9%) were obtained from Thermo Fisher Scientific (Hampton, NH) and Sigma-Aldrich. Acetic acid (≥99.7%), trichloroacetic acid (TCA) (≥99.0), dipotassium phosphate (K_2_HPO_4_) (≥60.0), and monopotassium phosphate (KH_2_PO_4_) (≥60.0) were obtained from Anachemia (Rouses Point, NYC). ISOP was obtained from ChromaDex (Irvine, CA) (98.3%) and Sigma-Aldrich (≥95.0%). Spectral grade dimethyl sulfoxide (DMSO) was obtained from Caledon (Georgetown, ON). High-purity nitrogen gas (N_2_), oxygen gas (O_2_), and carbon monoxide (CO) gas was obtained from Praxair (Danbury, CT).

[3′-*N*-^14^C-methyl]-Caffeine (specific activity 50–60 mCi/mmol) was obtained from American Radiolabeled Chemicals (St. Louis, MO). The methyl-^14^C labeled 8-MOP, specific activity of 40–60 mCi/mmol, was purchased from Vitrax Radiochemicals (Placentia, CA). Scintillation cocktail fluids were obtained from Perkin Elmer Life Sciences (Waltham, MA) and Amersham Biosciences (Piscataway, NJ). Ultrapure water was produced using a Millipore system (Billerica, MA) with a minimum resistivity of 16.0 MΩ cm at 25°C.

#### Human Liver Microsomes (HLMs)

Pooled HLMs were purchased from BD Gentest (Franklin Lakes, NJ; catalog number 452161 and lot numbers of 99268 and 18888) and GIBCO (Waltham, Massachusetts, catalog number HMMCPL and lot number PL050B).

### Caffeine Pharmacokinetics in Humans Before and After ARFP Extract Pre-treatment

#### Volunteer Eligibility and Selection

Male human volunteers, between 21 and 30 years old and living in the Metro Vancouver area, were recruited for the study. The volunteers had to meet specific eligibility criteria: not smoking, use medication, consume alcohol heavily or have any health concerns which may affect the test results. The volunteers were asked to avoid consuming caffeinated drinks and caffeine-containing foods for 12.0 h prior to and during the study period. The participants were also asked to refrain from consuming solid foods 3.0 h prior to the study. The study protocol was approved by the Simon Fraser University Office of Research Ethics with approval number 2012s0565 and registration number ISRCTN83028296.

#### Extraction of ARFP Products

The ARFP products were extracted as per our previous study (Alehaideb et al., 2017). An ARFP product was weighed accurately and powdered in a food processor. The powders were mixed with 600 ml of distilled water and boiled on a hot plate until half of the volume was evaporated. The aqueous extracts were filtered using a metal sieve.

#### Dosimetry of Furanocoumarin Mixtures

The dose measure of the furanocoumarin mixture in an ARFP extract was predicted using the concentration addition model (ATSDR, 2004; Law et al., 2017; Alehaideb et al., 2019):

$$Cmix=\overset{n}{\underset{i=1}{\sum }}\;Ci\;\times \;RPi$$

Where Cmix is the effect-based dose of the furanocoumarin mixture in ARFP extract; it is expressed in µM concentration equivalents of 8-MOP, Ci is the concentration of the *i*th furanocoumarin component in µM unit. The RPi (relative potency) is the IC_50_ of the *i*th furanocoumarin component relative to the IC_50_ of 8-MOP. The relative potency of a furanocoumarin bioactive represents an equally effective 8-MOP concentration for CYP 1A2 activity inhibition. Table 2 summarizes the weights and effect-based dose measures of the ARFP products in the present study.

**TABLE 2:** The effect-based dose measures of furanocoumarin mixtures in ARFP extracts.

Plant product name	Weight of plant product extracted	Effect-based dose of furanocoumarin mixture in water extract
	G	8-MOP equiv µM
*P. aniseum* seeds	10.0	2.4
*P. crispum* leaves	10.0	3.7
*A. pubescens* roots	12.0	8.9
*A. graveolens* seeds	10.0	16.4
*A. graveolens* flakes	10.0	28.3
*A. archangelica* roots	4.5	77.1
*R. graveolens* leaves	3.0	83.4
*C. monnieri* fruits	3.0	96.7
*A. archangelica* roots	9.0	179.4
*A. majus* seeds	6.0	559.6
*A. majus* seeds	12.0	913.3

#### Caffeine Pharmacokinetics in the Saliva/Plasma of Humans

##### Saliva Sampling After Caffeine Administration

This study adopted a non-randomized, single-blinded, and crossover design in which each subject acted as his own control (Sheriffdeen et al., 2019). Each volunteer was supplied with a study kit containing several caffeine tablets from AdremPharma WakeUps™ (Scarborough, ON), sampling vials, and instructions to conduct the study at home. In the baseline study, each volunteer received a single oral dose of two caffeine tablets (total 200 mg) and saliva samples (1–2 ml) were collected at 0.5, 1.0, 1.5, 2.0, 2.5, 3.0, 4.0, 5.0, 6.0, 7.0, 8.0, 12.0, and 24.0 h post-dosing. In the ARFP extract treatment study, the same group of volunteers was used after a 4 days washout period. In this study, the volunteers consumed an ARFP extract 3.0 h before taking the caffeine tablets. Saliva samples were collected at 0.5, 1.0, 1.5, 2.0, 2.5, 3.0, 4.0, 6.0, 8.0, 12.0, 24.0, 36.0, and 48.0 h post-dosing. All saliva samples were stored in the dark at freezing temperature until they were ready for extraction and HPLC-UV analysis.

##### Extraction of Saliva Samples

The saliva samples were thawed at room temperature, mixed by vortexing for 30 s, and centrifuged for 10 min at 4,000×*g*. Exactly 200.0 µl aliquot of the saliva sample was removed and spiked with 100.0 µl of the internal standard (ISTD) benzotriazole (50.0 μg/ml). The saliva samples were extracted once with 4.0 ml ethyl acetate by vortexing for 2.0 min and then centrifuged at 4,000×*g* for 5.0 min. The organic layer was removed and evaporated down to dryness, using a gentle stream of N_2_ gas. The residues were reconstituted in 150.0 µl mobile-phase of the HPLC. Exactly 100.0 µl aliquot of the solution was injected into the HPLC-UV system.

##### Quantifying Caffeine in Saliva Samples With HPLC-UV

Caffeine concentrations in saliva samples were determined using a modified HPLC procedure of Perera et al. (2010). Caffeine and the benzotriazole ISTD were separated by an Agilent Zorbax XDB reverse-phase C-18 column (250 × 4.6 mm, 5 µm particle size) at room temperature. Isocratic elution was performed using a solution of water: acetonitrile: acetic acid (80:19:1 v/v/v) at a flow rate of 1.5 ml/min. The UV detector was set at 280 and 580 nm was used as a reference wavelength. The total analysis time was 20.0 min. The caffeine concentrations in the saliva samples were determined using a multi-level calibration curve by plotting the caffeine/benzotriazole peak area ratio against the caffeine concentration. The range of the caffeine concentration used was from 0.11–13.63 μg/ml. The limit of detection and limit of quantification of the HPLC-UV method were 11.4 and 43.1 ng/ml, respectively.

##### Determination of Caffeine Pharmacokinetic Parameters

Caffeine concentrations in the saliva samples were first converted to plasma concentrations using a 0.79 correction factor (Fuhr et al., 1993). Caffeine concentration in the plasma was plotted against the sampling time of the pharmacokinetic study with the Prism GraphPad software (San Diego, CA). The following pharmacokinetic parameters were determined: the T_max_ (time to reach peak plasma level) and C_max_ (concentration of peak plasma level) were determined by visual inspection. The AUC_0-inf_ (area under plasma concentration-time curve from zero to infinity) was determined by non-compartmental analysis using the PK Solver software (Zhang et al., 2010). The AUC_0-inf_ was determined using the log-linear trapezoidal rule from dosing time to last time point and extrapolated to infinity by dividing the last concentration with the elimination rate constant. The AUC ratio was calculated by dividing the AUC_0-inf_ after ARFP extract pretreatment with the AUC_0-inf_ before ARFP extract pretreatment.

### Inhibition of CYP 1A2-Mediated Caffeine-*N* ^3^-Demethylation *in vitro*

#### Quantitation of CYP 1A2 Inactivity

The caffeine-*N*-demethylase assay was conducted with [3′-*N*-^14^C-methyl]-caffeine according to Bloomer et al. (1995) with modification by Sheriffdeen et al. (2019). Briefly, the incubation contained non-labeled caffeine (82.0 µm), ^14^C-labeled caffeine (0.2 µCi), a furanocoumarin inhibitor (see concentrations in *IC*_*50*_ *Determination* and *Pre-incubation Time- and Furanocoumarin Concentration-dependent Inhibition of Caffeine Metabolism in vitro*), NADPH (1.34 mm), and potassium phosphate buffer (50.0 mm, pH 7.4) in a final volume of 200.0 µl. DMSO was used to dissolve caffeine and the furanocoumarin inhibitor; less than 1% incubation volume of DMSO was found to have no significant effect on caffeine 3-*N*-demethyation activity. Incubation was conducted at 37°C in a metabolic incubator with a 60 cycles/min shaking rate. At the conclusion of 10 min incubation, the reaction was terminated by the addition of 10% TCA solution (50.0 µl). The incubation mixture was centrifuged at 4,000×*g*. An aliquot (300.0 µl) of the supernatant was applied to a 3.0 ml Sigma-Aldrich Superclean ENVI-Carbon solid phase extraction (SPE) tube (0.25 g, 80–100 mesh). Demethylated metabolites of caffeine (i.e., ^14^C-formaldehyde and ^14^C-formic acid) were eluted from the SPE tube with 2.0 ml of water. The eluant was collected into a scintillation vial. After the addition of 15.0 ml scintillation cocktail, the radioactivity in the vial was counted in a liquid scintillation counter. The results were expressed as residual counts of the control incubation.

#### IC_50_ Determination

*In vitro* caffeine-*N*-demethylase activity was determined with various concentrations of a furanocoumarin inhibitor: 0.01–11.12 µm for 8-MOP; 0.06–7.10 µm for 5-MOP; and 0.01–38.45 µm for ISOP. CYP 1A2 activity inhibition was plotted against log furanocoumarin concentration after normalizing the inhibition data from 0–100%. The resulting inhibition-concentration curves were fitted separately to a four-parameter logistic function to determine the IC_50_ values. Curve fitting was performed using the GraphPad Prism version 5.04 (GraphPad Software, San Diego, CA). The IC_50_ represented the concentration of the furanocoumarin chemical to evoke a half maximal inhibition of the CYP 1A2 activity.

#### Pre-incubation Time- and Furanocoumarin Concentration-dependent Inhibition of Caffeine Metabolism *in vitro*

These studies were conducted in two steps: pre-incubation with a furanocoumarin inactivator followed by dilution and incubation to measure the caffeine metabolizing activity. In the pre-incubation step, HLMs (1.2 mg/ml) were pre-incubated with different furanocoumarin concentrations in the presence of NADPH for various time periods at 37°C. The pre-incubation times of the studies were: 0.5, 1.0, 1.5, and 2.0 min for 8-MOP and 5-MOP, and 0.5, 1.0, 2.0, and 3.0 min for ISOP. The furanocoumarin concentration ranges of the studies were: 1.07–17.14 µm for 8-MOP. 1.13–18.07 µM for 5-MOP, and 0.47–15.02 for ISOP. In the final incubation step, 32.0 µl of the pre-incubation mixture was transferred to an incubation vial containing fresh NADPH (1.0 mm), non-labeled caffeine (6.4 µm), and ^14^C-caffeine (0.2 µCi). The final incubation volume was 320.0 µl. At the conclusion of a 10 min incubation, the reaction was stopped by the addition of a 10% TCA solution. The incubation mixture was extracted by a SPE tube, and the radioactivity in the eluant was counted in a liquid scintillation counter as described earlier.

The observed *in vitro* rate constant (k_obs_) was calculated using the slopes of linear regression analyses from natural logarithm (Ln) percentage remaining activity against pre-incubation time plots. The maximum inactivation rate constant (k_inact_) was calculated using the non-linear regression analyses of plots of the inactivator concentrations against k_obs_ values. The inactivator concentration (K_I_) was extrapolated at 50% k_inact_ value. The plotting was performed using the GraphPad Prism version 5.04 (GraphPad Software, San Diego, CA).

#### Irreversible Binding of ^14^C-8-MOP-Derived Radioactivity to HLMs

These studies were conducted as per Jollow et al. (1973) with modification (Law and Chakrabarti, 1983; Law and Meng, 1996). The complete or control incubation mixture consisted of HLMs (1.0 mg), ^14^C-8-MOP (0.2 µCi), potassium phosphate buffer (50.0 mm, pH 7.4), and NADPH (1.0 mm) in a 1.34 ml final incubation volume. The incubation mixture was pre-warmed at 37°C for 5.0 min and the reaction was started with the addition of HLMs. After a 10 min incubation, the reaction was terminated by the addition of ice-cold 10% TCA solution. Unchanged ^14^C-8-MOP and water-soluble metabolites in the incubation were washed off repeatedly with three cycles each of methanol, acetonitrile, and acetone. Our preliminary studies indicated that the three-cycle solvent washes were sufficient to remove all unbound radioactivity from the precipitates of HLMs. The ^14^C bound to the protein precipitates was determined using a liquid scintillation counter after the addition of 15.0 ml scintillation cocktail. The effects of different incubation conditions on ^14^C-adducts formation were studied by omitting NADPH cofactor, depleting O_2_, adding CO or using heated HLMs in the complete incubation mixture.

### Statistical Analysis

The data were analyzed using the Student’s paired *t*-test, two-tailed and 95% confidence interval using Microsoft Excel. The difference between two comparable data sets were considered significant when *p* ≤ 0.05.

## Results

### Caffeine and Furanocoumarin Bioactive in Saliva Samples

**FIGURE 2:**
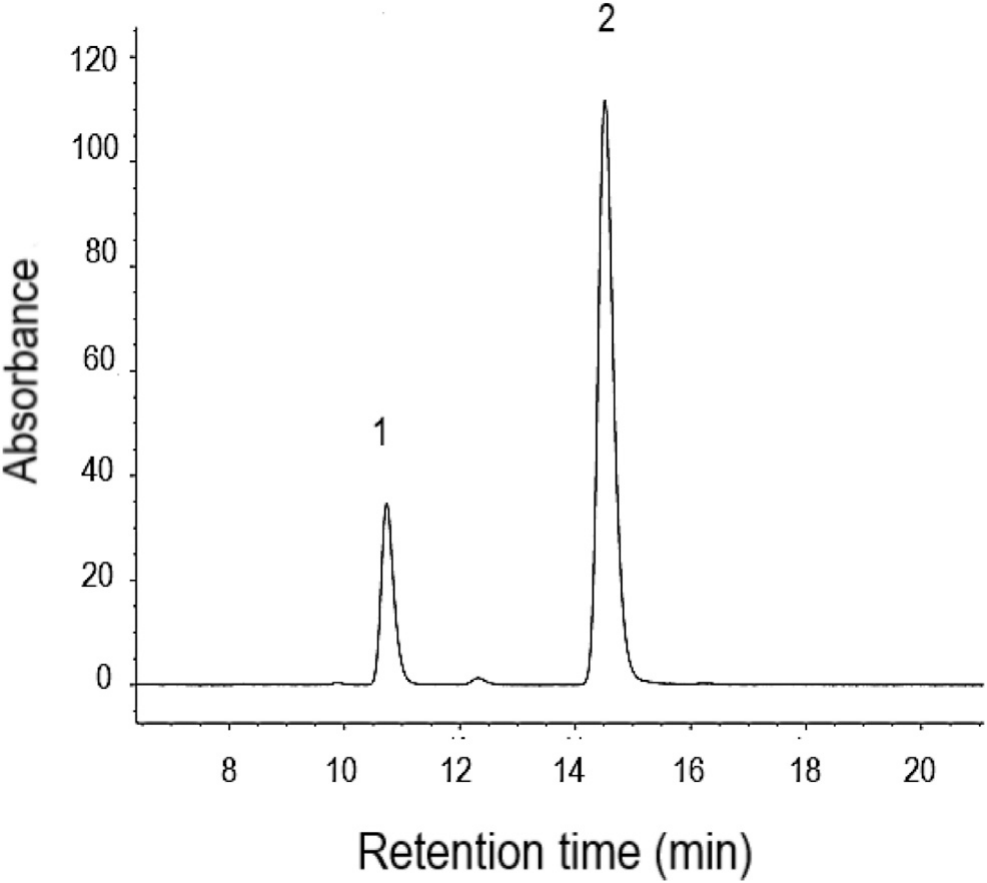
The separation of caffeine (1) and benzotriazole (2), the internal standard, in saliva extract using isocratic method by HPLC with UV. The retention time for caffeine and benzotriazole is 10.7 and 14.5 min, respectively.

Figure 2 shows a typical HPLC chromatogram of caffeine and benzotriazole ISTD in the saliva extract. Little or no caffeine was detected in the saliva samples before the human volunteers consumed the caffeine tablets. Neither furanocoumarin bioactive nor unknown phytochemicals were found in the saliva samples after the volunteers were treated with an ARFP extract.

### Caffeine Pharmacokinetics in Human Volunteers Before and After Pre-treatment With an ARFP Extract

**FIGURE 3:**
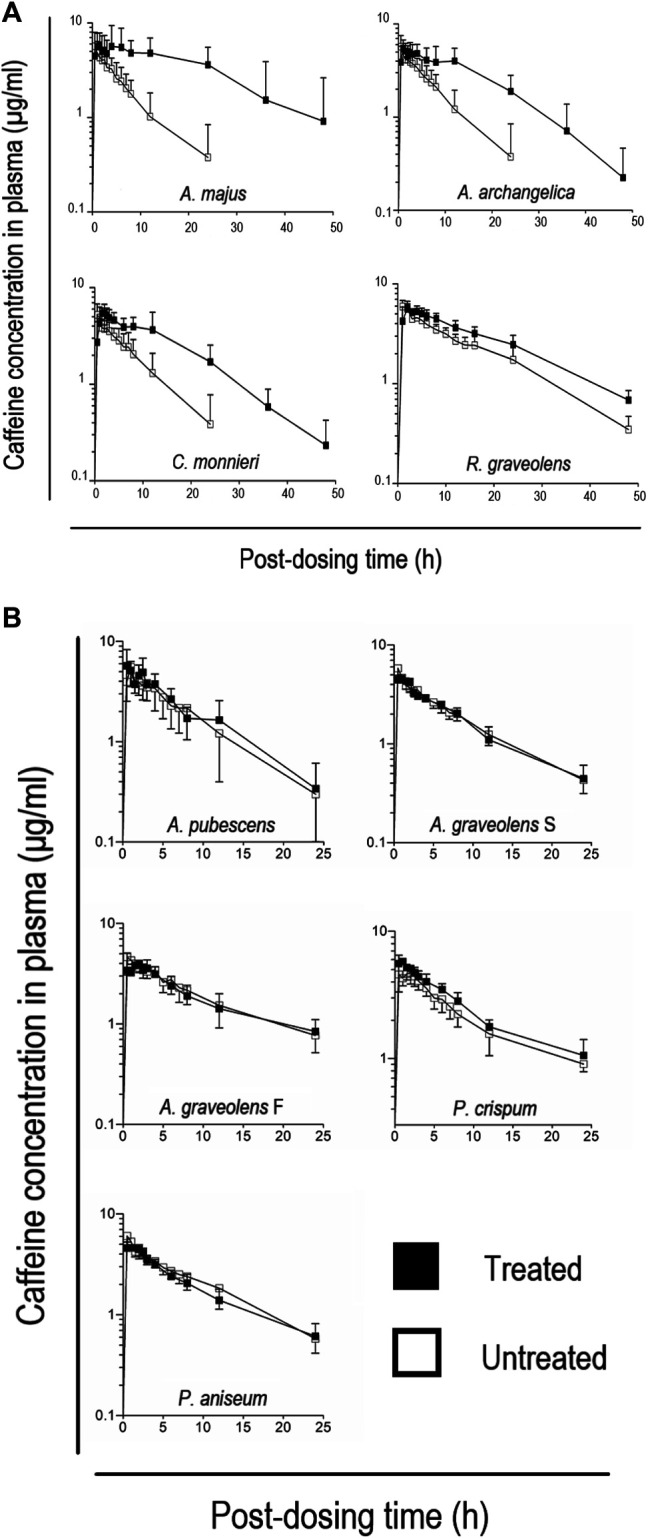
**(A)** Time course of caffeine concentration in the plasma of volunteers before and after pre-treatment with an extract of *A. majus, A. archangelica, C. monnieri,* and *R. graveolens*. Each point is plotted as mean ± SD. Empty squares represent caffeine concentrations in volunteers with no ARFP extract pre-treatment. Filled squares represent caffeine concentrations in volunteers with ARFP extract pre-treatment 3.0 h before receiving the caffeine tablets. **(B)** Time course of caffeine concentrations in the plasma of volunteers before and after pre-treatment with an extract of *P. aniseum, P. crispum, A. pubescens, A. graveolens* seeds*,* and *A. graveolens* flakes. The point is reported as mean ± SD. Empty squares represent caffeine concentrations in volunteers with no ARFP extract pre-treatment. Filled squares represent caffeine concentrations in volunteers with ARFP extract pre-treatment 3.0 h before receiving the caffeine tablets.

Figure 3A displays the time course of caffeine concentration in the plasma of human volunteers before and after pre-treatment with an aqueous extract of *A. majus* seeds, *A. archangelica* roots, *C. monnieri* fruits or *R. graveolens* leaves. Caffeine concentrations in the plasma were found to increase significantly after each ARFP extract pre-treatment as seen in Figure 3A. Table 3 summarizes the pharmacokinetic parameters of the concentration-time curves. Pre-treatment of humans with *A. majus* seeds extract significantly increased the AUC_0-inf_ of caffeine by 432%, the T_max_ from 0.8 to 2.4 h, and the C_max_ by 16.6%. *A. archangelica* roots extract pre-treatment increased the AUC_0-inf_ of caffeine by 232%, the T_max_ from 0.8 to 1.3 h, and no change for C_max_ value. If 2x weights of *A. majus* seeds and *A. archangelica* roots were used in the extraction (Table 3 bottom rows), the AUC_0-inf_ of caffeine increased by 577 and 267%, respectively. For *A. archangelica* 2x, the double-dose delayed T_max_ and increased C_max_ about 47%. Not much change for *A. majus* seeds double-dose in comparison to the less dose. *C. monnieri* fruits extract increased the AUC_0-inf_ of caffeine by 220%, T_max_ from 0.7 to 1.8 h but had no effect on the C_max_ whereas *R. graveolens* leaves extract pre-treatment increased the AUC_0-inf_ of caffeine by 135%, the T_max_ from 0.6 to 1.0 h, and the C_max_ by 12%. Interestingly, the clearance (CL) of caffeine was decreased significantly after pre-treating the volunteers with these plant extracts. Thus, pre-treatment of humans with one of these 4 ARFP extracts resulted in an increase in caffeine AUC_0-inf_ with a concomitant decrease in CL, a finding which clearly showed these ARFP extracts were potent inhibitors of caffeine metabolism.

**TABLE 3:** Caffeine pharmacokinetic parameters in human volunteers before and after pre-treatment with the aqueous extract of *A. majus, A. archangelica, C. monnieri,* or *R. graveolens*. The ±SD of the data is omitted for better display and comparison.

Plant product name	*n* ^a^	T_max_		C_max_		AUC_0-inf_		CL	
		H		µg/ml		µg/ml*h		ml/min	
		UT^b^	T^b^	UT	T	UT	T	UT	T
*A. majus* seeds	6	0.8	2.4	5.4	6.3^c^	40.4	174.6^d^	95.6	21.7^d^
*C. monnieri* fruits	5	0.7	1.8^d^	6.0	5.9	46.1	101.8^c^	85.2	40.5^d^
*R. graveolens* leaves	6	0.6	1.0	6.6	7.4	50.9	68.6^c^	82.1	60.4
*A. archangelica* roots	6	0.8	1.3^d^	5.4	5.4	43.3	100.3^d^	84.9	44.8^d^
*A. majus* seeds^e^	2	0.5	2.5	5.0	6.8	34.2	197.4^c^	116.7	17.1
*A. archangelica* roots^e^	4	0.8	2.8	5.1	7.5	46.5	124.3^d^	90.5	28.8

^a^ *n* is the number of human volunteers in the study.

^b^ UT = untreaed human volunteers, T = human volunteers pretreated by an aqueous extract.

^c^ Significantly different to untreated human volunteer (0.01 < *p* ≤ 0.05).

^d^ Significantly different to untreated human volunteer (*p* ≤ 0.01).

^e^ Double-dose of the **A. majus** seeds and **A. archangelica** roots presented before.

Figure 3B shows the time course of caffeine concentration in the plasma of volunteers before and after they were pre-treated with an extract of *A. pubescens* roots, *A. graveolens* seeds, *A. graveolens* flakes, *p. aniseum* seeds, or *p. crispum* leaves. Pre-treatment of human volunteers with the remaining 5 ARFP extracts did not change the concentration-time profiles of caffeine significantly. Table 4 summarizes the pharmacokinetic parameters of the caffeine concentration-time curves in these studies. Pre-treatment of humans with *A. graveolens* seeds extract increased the AUC_0-inf_ of caffeine by 12%, the T_max_ from 0.7 to 1.2 h, but the C_max_ was reduced by 8.2% but the increase in AUC_0-inf_ was statistically insignificant compared to the untreated volunteers. The pre-treatment with *A. graveolens* flakes extract increased the mean T_max_ of caffeine from 0.8 to 1.5 h, but had no effect on the C_max_ and AUC_0-inf_. The pre-treatment with *P. aniseum* seeds extract increased the T_max_ of caffeine from 0.6 to 1.1 h, reduced the C_max_ by 16.9%, and had no effect on the AUC_0-inf_. The pre-treatment with *P. crispum* leaves increased the AUC_0-inf_ of caffeine by 27.5% but the increase was statistically insignificant. *P. crispum* leaves pre-treatment also increased the C_max_ of caffeine by 5.0% but had no effect on the T_max_. Interestingly, treatment with *A. pubescens* roots decreased AUC_0-inf_ by 30.0% and C_max_ by 40% but both were found not significant (*p* > 0.05). However, treatment with *A. pubescens* roots increased Tmax from 2.4 to 3.1 h. In contrast, the CL of caffeine in these studies was decreased after ARFP pre-treatment. Thus, none of the pharmacokinetic parameters, with the exception of CL for *A. pubescens* roots treatment (*p* value 0.04), was changed significantly after the volunteers were pre-treated with these 5 ARFP extracts.

**TABLE 4:** Caffeine pharmacokinetic parameters of human volunteers before and after pre-treatment with the water extract of *P. aniseum*, *P. crispum*, *A. pubescens*, *A. graveolens* seeds, or *A. graveolens* flakes. The ± SD of the data is omitted for better display and comparison.

Plant product name	*n* ^a^	T_max_		C_max_		AUC_0-inf_		CL	
		H		µg/ml		µg/ml*h		ml/min	
		UT^b^	T^b^	UT	T	UT	T	UT	T
*A. graveolens* flakes	4	0.8	1.5	5.4	5.4	50.1	52.2	73.5	67.7
*A. graveolens* seeds	6	0.7	1.2	6.1	5.6	42.9	48.2	91.3	74.7
*A. pubescens* roots	5	2.4	3.1	5.0	2.9	33.5	23.6	160.3	141.4
*P. crispum* leaves	4	1.2	1.1	6.0	6.3	53.0	73.1	78.0	56.3
*P. aniseum* seeds	6	0.6	1.1	6.5	5.4	55.8	54.0	77.9	76.4

^a^ *n* is the number of human volunteers in the study.

^b^ UT = untreaed human volunteers, T = human volunteers pretreated by an aqueous extract.

**FIGURE 4:**
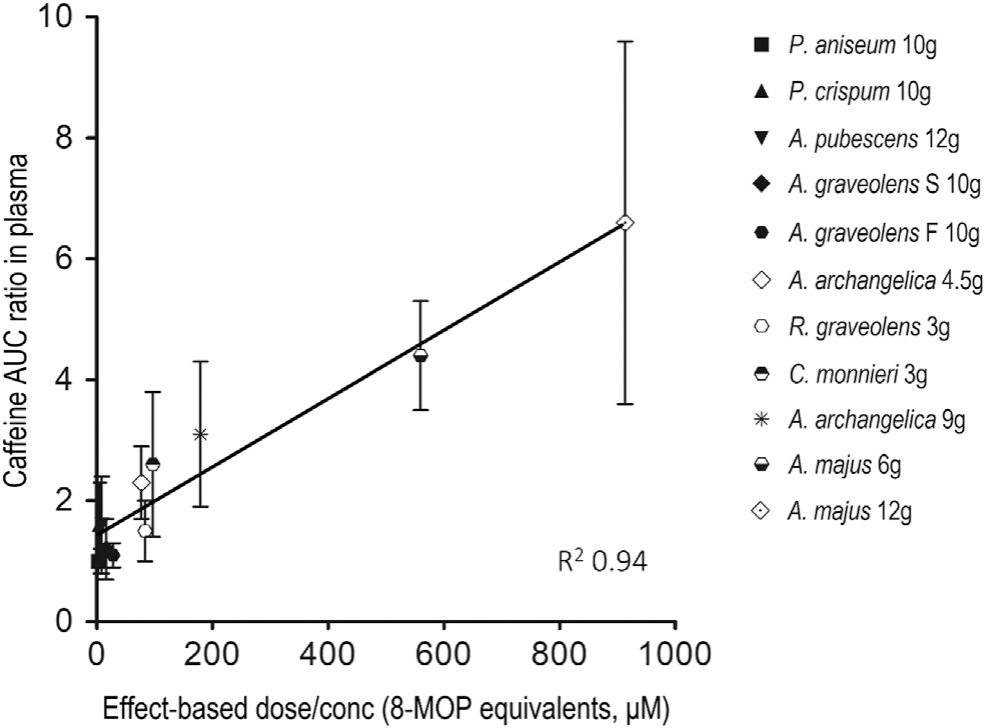
Correlation between the area-under-the-concentration-time-curve (AUC) ratio of caffeine and the effect-based dose measure of the furanocoumarin mixture in ARFP extract. The data represent mean ± SD of caffeine AUC ratio with different effect-based dose measures in the ARFP extracts.

Figure 4 depicts the relationship between caffeine AUC ratio and the effect-based dose measure of the 8-MOP, 5-MOP and ISOP mixture in the ARFP extract. Caffeine AUC ratio correlated linearly with the effect-based dose of the furanocoumarin mixture (*R* ^2^ = 0.94). Thus, *A. majus* seeds, *A. archangelica* roots, *C. monnieri* fruits, and *R. graveolens* leaves, which had large effect-based dose measures and were potent caffeine metabolism inhibitors, were located at the upper part of the straight line. The remaining ARFP products which were less potent inhibitors and had small effect-based dose measures, were located at the lower part of the straight line. In other words, the extent of CYP 1A2 inhibition is determined by the additive combination effects of just 3 furanocoumarin bioactive (i.e., 8-MOP, 5-MOP, and ISOP) in the ARFP extract.

### *In Vitro* Inactivation of Microsomal Caffeine-*N*-Demethylation Activity by Individual Furanocoumarin Chemicals

**FIGURE 5:**
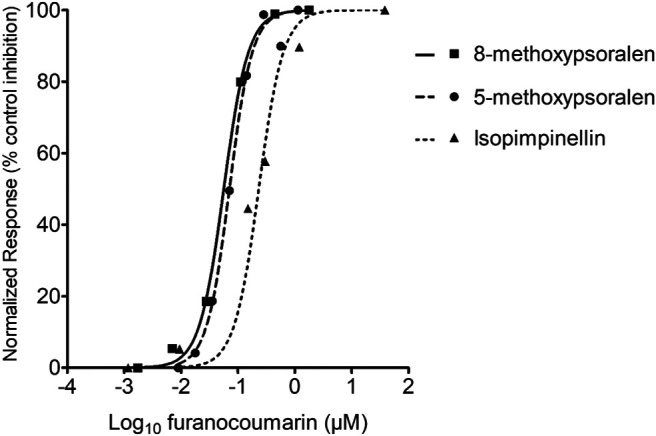
Effects of furanocoumarin concentration on CYP 1A2 inactivation *in vitro*. The results are presented as the mean of at least three incubations. The ±SD of the data is omitted for better display and comparison. The IC_50_ was determined from the concentration-inhibition curve using non-linear regression analysis.

Little or no caffeine was metabolized by the HLMs if NADPH were omitted from the incubation (data not shown). Caffeine-*N*-demethylase activity was significantly inhibited when 8-MOP, 5-MOP, or ISOP was added to the incubation. Also, caffeine-*N*-demethylase activity appeared to decrease with an increasing furanocoumarin concentration in the incubation. Figure 5 shows the inhibition-log_10_ concentration curves of 8-MOP, 5-MOP, and ISOP on caffeine metabolism. The inhibition-concentration curves were parallel to one another, indicating the linear furanocoumarins and caffeine were bound to similar metabolic sites on the CYP 1A2 isozyme. The IC_50_ of 8-MOP, 5-MOP, and ISOP on caffeine metabolism were determined to be 0.09 ± 0.05, 0.13 ± 0.11, and 0.29 ± 0.22 µm (mean ± standard deviation (SD)), respectively. Thus, the inhibition potency of the furanocoumarin bioactive decreased in the order of 8-MOP > 5-MOP > ISOP. The IC_50_ values were used to calculate a relative potency (or inhibitory equivalence factor) for the furanocoumarin bioactive in the ARFP extracts. The inhibitory equivalence factor for 8-MOP, 5-MOP, and ISOP were 1.00, 0.69, and 0.31, respectively, assuming the 8-MOP value was equal to 1.00. The effect-based dose for the furanocoumarin mixture in an ARFP extract was predicted using the concentration addition model. Table 2 summarizes the ARFP product weights used in the extraction and the corresponding effect-based dose measures of the furanocoumarin mixture in the present study.

**FIGURE 6:**
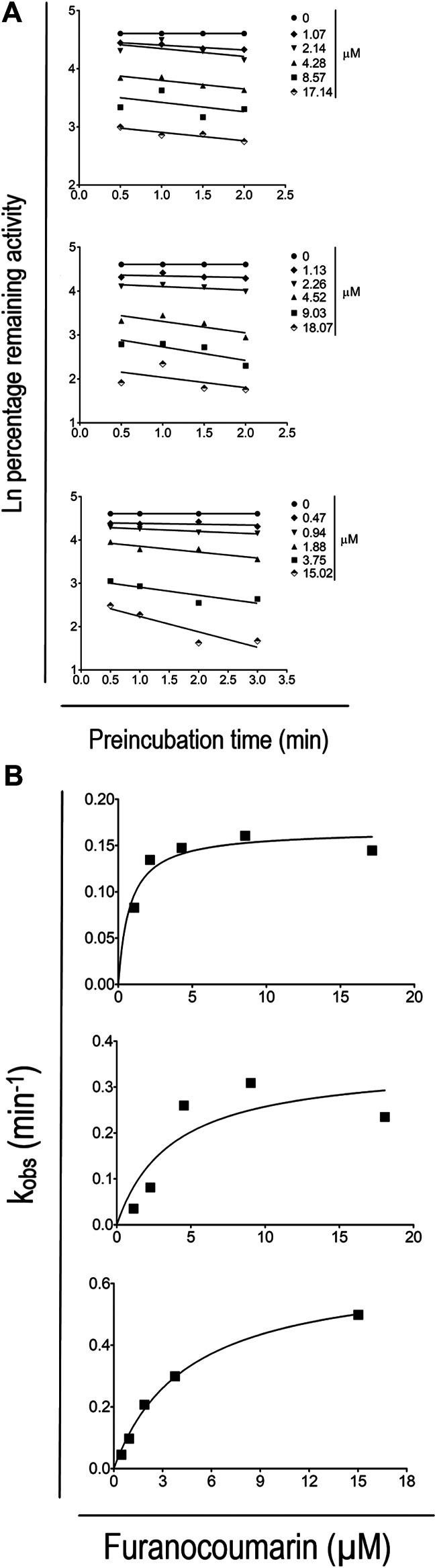
**(A)** Time- and concentration-dependent inactivation of CYP 1A2 activity by 8-MOP **(upper)**, 5-MOP **(middle)** and ISOP **(lower)**. The results are presented as the mean of at least three independent incubations. The ±SD of the data is omitted for better display and comparison. **(B)** Maximum inactivation rate of CYP 1A2 activity by 8-MOP **(upper)**, 5-MOP **(middle)** and ISOP **(lower)**. The results are presented as the mean of at least three independent incubations. The ±SD of the data is omitted for better display and comparison.

Figure 6A shows the effects of pre-incubation time and furanocoumarin concentration on CYP 1A2 inactivation. The degree of caffeine metabolism inhibition was time- and concentration-dependent, indicating involvement of the irreversible inhibition mechanism. Figure 6B shows the non-linear relationship between k_obs_ and the concentration of individual furanocoumarin in these studies. The K_I_ values for 8-MOP, 5-MOP, and ISOP were estimated at 0.78 ± 0.32, 3.73 ± 3.66, and 4.48 ± 0.56 µm, respectively. The estimated k_inact_ values were 0.17 ± 0.01, 0.35 ± 0.12, and 0.65 ± 0.03 min^−1^, respectively.

The proposed irreversible inhibition mechanism was studied further with ^14^C-8-MOP metabolism studies. ^14^C-8-MOP biotransformation in NADPH-fortified HLMs resulted in the formation of ^14^C-adducts which were studied using different incubation conditions. Table 5 shows little or no ^14^C-adducts were formed when boiled HLMs were used or NADPH and O_2_ were omitted in the incubation. An increase of the CO concentration in the incubation decreased the amounts of ^14^C-adducts formed. These results indicated that ^14^C-8-MOP reactive metabolite(s) was formed and bound irreversibly to the HLMs during ^14^C-8-MOP biotransformation.

**TABLE 5:** The effects of different incubation conditions on the amounts of irreversibly bound radioactivity formed in human liver microsomes.

Incubation mixture	Irreversible bound 8-MOP-derived^14^C^a^
	(pmol per mg protein)
Control	3.67 ± 0.39
Boiled microsomes (+90°C)	0.43 ± 0.03^b^
Without NADPH (-NADPH)	0.51 ± 0.14^b^
Without oxygen (+N_2_)	2.66 ± 0.44^b^
With carbon monoxide (+CO)	2.58 ± 1.06^b^

^a^ Results are expressed as mean ± SD, three independent incubation replicas were performed for each experiment.

^b^ Significantly different to control (p ≤ 0.05).

## Discussion

Our results show all nine tested ARFP products are able to inhibit caffeine metabolism in humans to some extent (Figures 3A,B; Tables 3, 4). These findings are consistent with previous reports that caffeine and linear furanocoumarins are metabolized by the same hepatic CYP 1A2 isozyme. The *A. majus*, *A. archangelica*, *C. monnieri*, and *R. graveolens*, are the most potent inhibitors among the tested products since they can alter the pharmacokinetics of caffeine in humans by increasing the AUC ratio of caffeine with a concomitant decrease in CL (Table 3).

A range of AUC values (33.5–55.8 μg/ml*h) is observed in the untreated or control volunteers in our study (Tables 3, 4). The wide AUC range values probably are due to the *inter*- and *intra*-individual differences in caffeine pharmacokinetics (Tian et al., 2019). As such, a comparison of AUC values between our and other studies would render additional confidence in our results. The mean ± SD caffeine AUC value in healthy volunteers after receiving a 5 mg/kg dose is 60.8 ± 23.7 μg/ml*h (Kamimori et al., 1995). This is very close to the high values in our AUC range (Tables 3, 4)). In contrast, an AUC value of 28.5 ± 14.9 μg/ml*h is reported for 12 human volunteers after receiving 167 mg of caffeine orally (Fuhr et al., 1993). This is comparable to the low values in our AUC range.

The AUC ratio of caffeine in human volunteers correlates linearly with the effect-based dose of the furanocoumarin mixture administered (Figure 4). The linear dose-inhibition relationship indicates that the degree of caffeine metabolism inhibition is dependent mainly on the additive combination effects (or the effect-based dose) of three linear furanocoumarins (i.e., 8-MOP, 5-MOP, and ISOP) in the ARFP products although other known and/or unknown phytochemicals also may participate in caffeine metabolism inhibition. For example, the apigenin in *P. crispum* (Meyer et al., 2006) and the osthole in *A. pubescens* (Ko et al., 1989) have been shown to be inhibitors of CYP 1A2 activity (Peterson et al., 2006; Yang et al., 2012). However, because of the linear dose-inhibition relationship and the high correlation coefficient, it is unlikely apigenin, osthole or an unknown phytochemical plays an important role in caffeine metabolism inhibition.

The inhibitory effects of ARFP products appear to last for a relatively long time since the ARFP extract is administered to the human volunteers 3.0 h prior to caffeine consumption (see *Saliva Sampling After Caffeine Administration*.). The long inhibitory effects of ARFP products are consistent with the following studies: Firstly (Mays et al., 1987), have shown humans dosed with methoxsalen (or 8-MOP) 1.0 h before are still able to inhibit caffeine metabolism. Secondly, the furanocoumarin bioactive in ARFP products can destroy CYP 1A2 isozyme because they are irreversible inhibitors. Since the only means for the CYP 1A2 to regain its activity is by isozyme re-synthesis, it would take a long time. Thirdly, the timing of ARFP inhibition and the T_max_ of individual furanocoumarins overlap one another. For example, the mean T_max_ of 8-MOP in Psoralen and Ultraviolet A (PUVA) patients is reported to range from 0.6 ± 0.2 h to 2.3 ± 0.9 h (Siddiqui et al., 1984). In another study, the mean T_max_ of 8-MOP in PUVA patients ranges from 0.9 ± 0.4 h to 2.0 ± 0.6 h (Walther et al., 1992). The T_max_ of 5-MOP in healthy humans and PUVA patients are 3.0 ± 0.6 h (Stolk et al., 1981) and 2.8 ± 0.8 h (Shephard et al., 1999), respectively. Although we are unable to find the T_max_ of ISOP in humans, the T_max_ of ISOP in rats after receiving an oral dose of the *Toddalia asiatica* L (Lam) and *C. monnieri* herbal products are shown to be 1.3 ± 0.3 h (Liu et al., 2012) and 1.1 ± 0.3 h (Li et al., 2014), respectively.

The inhibition-concentration curves for pure 8-MOP, 5-MOP, and ISOP chemicals are parallel to one another (Figure 5). Villeneuve et al. (2000) have shown that parallelism in the inhibition-concentration curves is a pre-requisite to determine an accurate relative potency for the CYP inhibitors. In the present study, the IC_50_ of 8-MOP, 5-MOP, and ISOP on caffeine metabolism are 0.09, 0.13, and 0.29 µm, respectively. In contrast, the IC_50_ of 8-MOP, 5-MOP, and ISOP on ethoxyresorufin-*O*-deethylase activity are 9.6, 2.3, and 1.9 µm, respectively in mice liver microsomes (Cai et al., 1993). The IC_50_ of 8-MOP on 7-ethoxycoumarin deethylase and benzo(a)pyrene hydroxylase activities are about 25 µm in HLMs (Tinel et al., 1987). An explanation for the discrepancy in results between our and other studies is not readily available but is likely related to the different animal species, amounts of microsomal proteins, choice of probe substrates, and detection methods used in the studies. Nevertheless, because of the parallel inhibition-concentration curves, we are confident that the IC_50_ and the derived relative potency are determined accurately.

*In vitro* CYP 1A2 inactivation by individual furanocoumarins is pre-incubation time- and furanocoumarin concentration-dependent (Figures 6A,B) which are the characteristics of the irreversible or mechanism-based inhibition mechanism. Our proposed inhibition mechanism is consistent with the following publications: 1) the ISOP is a mechanism-based inhibitor of human CYP 1A2 isozyme with K_I_ and k_inact_ values of 1.2 µm and 0.34 min^−1^ respectively (Kang et al., 2011), 2) the 8-MOP is a time-dependent, suicide inhibitor for the CYP enzymes of mice and rats (Folin-Fortunet et al., 1986; Letteron et al., 1986; Mays et al., 1990). Also, 8-MOP is a time-dependent inhibitor of CYP 2A6 enzyme in humans (Koenigs et al., 1997), 3) the 5-MOP is capable of inactivating CYP enzymes in rats (Fuhr et al., 1993), and 4) (Folin-Fortunet et al., 1986) have proposed a dual mechanism of competitive inhibition and irreversible inhibition with the latter being the dominant mechanism to explain CYP enzyme inactivation by methoxsalen in the rat.

The removal of NADPH or the reduction of O_2_ level in the incubation results in decreased formation of ^14^C-adducts (Table 5). These results indicate ^14^C-8-MOP biotransformation is mediated by an oxidation reaction of HLMs and ^14^C-adducts are the by-products of ^14^C-8-MOP metabolism. We hypothesize the formation of ^14^C-adducts to involve the following steps: the furan ring of ^14^C-8-MOP is first oxidized by CYP 1A2 to an electrophilic epoxide intermediate(s) which then binds irreversibly to the CYP 1A2-substrate complex and inactivates the CYP 1A2 isozyme itself. Our hypothesis is supported by the reports that 8-MOP is metabolized by epoxidation of the furan ring in dogs (Kolis et al., 1979), and furan-containing chemicals are metabolized by opening of the fused ring (Sahali-Sahly et al., 1996). In other words, furanocoumarin metabolism leads to CYP 1A2 isozyme destruction and a reduction of caffeine-metabolizing activity in humans. This has been termed “suicide enzyme inhibition” (Letteron et al., 1986; Clewell and Andersen, 1987; Mays et al., 1990). Once the CYP 1A2 isozyme is deactivated, its activity can only be regained by isozyme re-synthesis. The time required to regenerate CYP 1A2 activity after its destruction is long and this explains why the ARFP products still are able to inhibit caffeine metabolism in humans treated with a single oral dose of ARFP extract 3.0 h before.

## Conclusion

Pre- or co-administration of caffeine and ARFP products may result in CYP 1A2 inactivation, caffeine overdose, and serious health consequences. As such, care must be exercised when furanocoumarin-rich plant products are co-administered with drugs that are substrates of the CYP 1A2 isozyme. Our studies also support the development of regulations for proper labeling of natural health products and functional foods that contain inhibitors of CYP enzymes.

## Data Availability

The original contributions presented in the study are included in the article/Supplementary Material, further inquiries can be directed to the corresponding author.

## Abbreviations

5-MOP, 5-methoxypsoralen; 8-MOP, 8-methoxypsoralen; ISOP, isopimpinellin; ARF, Apiaceae and Rutaceae families; AUC, area-under-the-concentration-time-curve; AUC0-inf, AUC from time zero time to infinity; CL, oral clearance; Cmax, maximum concentration; CO, carbon monoxide; CYP, cytochrome 450P; DMSO, dimethyl sulfoxide; HLMs, human liver microsomes; HPLC-UV, high-performance liquid chromatography and ultra-violet detector; IC_50_, inhibition concentration at 50%; ISTD, internal standard; KI, irreversible inhibition constant; kinact, maximum inactivation rate constant; kobs, observed rate constant; Ln, natural logarithm; NADPH, β-nicotinamide adenine dinucleotide phosphate hydrogen; N_2_, nitrogen gas; O_2_, oxygen gas; PUVA, psoralen and ultra-violet A; SPE, solid-phase extraction; SD, standard deviation; TCA, trichloroacetic acid; Tmax, time at maximum concentration.
